# Influence of Biopolymer Carrageenan and Glycerine on the Properties of Extrusion Printed Inks of Carbon Nanotubes

**DOI:** 10.3390/polym10101148

**Published:** 2018-10-15

**Authors:** Mohammed Almoiqli, Ali Aldalbahi, Mostafizur Rahaman, Periyasami Govindasami, Shaykha Alzahly

**Affiliations:** 1 Nuclear Sciences Research Institute, King Abdulaziz City for Science and Technology, Riyadh 11442, Saudi Arabia; almoiqli@kacst.edu.sa; 2 Department of Chemistry, College of Science, King Saud University, Riyadh 11451, Saudi Arabia; pkandhan@ksu.edu.sa; 3 King Abdullah Institute for Nanotechnology, King Saud University, Riyadh 11451, Saudi Arabia; shaykha.alzahly@hotmail.com

**Keywords:** Extrusion printing inks, contact angle, electrical conductivity, morphology

## Abstract

This article focuses on the preparation of extrusion printing composite inks of multiwall carbon nanotube (MWNT) dispersed separately in iota-carrageenan (IC) and glycerine (G) solution. Both composites (IC-MWNT and G-MWNT) showed shear-thinning behavior when their flow characteristics were tested. Conductive solid tracks/patterns of both printed composite inks were deposited on glass slide, PET (polyethylene terephthalate) sheet, and IC gel films substrates. The conductive patterns were characterized with microscopy, scanning electron microscopy (SEM), and profilometer. Moreover, their contact angle and electrical conductivity were measured. Profilometry showed that increased number of extruded layers gave increased cross-sectional area. SEM study showed that printing ink is embedded into the surface of IC film, discontinuous on glass slide and smoother on PET sheet. Conductivity of IC-MWNT track was 9 ± 1 S/m and that of G-MWNT was 2942 ± 84 S/m on glass substrate of one layer thick. This is because fewer carbon nanotubes (CNT) are present in G-MWNT track as confirmed by SEM study. The nature of substrate also affects the conductivity of printed patterns. The impressive result of conductivity of printed patterns of composite inks can make them useful for bioelectronic application.

## 1. Introduction

Carbon nanotube (CNT) has versatile unique properties in electrical, thermal, optical, and mechanical fields and is, thus, a potential material to prepare ink for high speed printing. There are two main printing methods, inkjet and extrusion [1,2,3,4]. Extrusion printing process involves pushing high-viscosity inks under constant pressure through a small nozzle of a syringe onto a moving substrate [3,5]. Compared to inkjet printing, extrusion printing creates patterns which can reliably deposit large volumes through cheap and replaceable parts [6]. This method can also be applied to manufacture three-dimensional structures and to embed material into substrates [3,7]. Unlike inkjet printing, extrusion printing allows for the patterning of high-viscosity inks [3]. Very careful control of the flow properties, surface tensions and wettability of the inks is required to successfully produce a high-quality extruded material [8].

In recent studies it has been shown that both inkjet and extrusion printing methods were used to prepare conducting tracks by depositing a conducting polymer, poly(3,4-ethylendedioxythiophene)/poly(sodium4-styrene-sulfonate), onto a chitosan biopolymer substrate [3]. In that work, a highly viscous paste was used for the syringe extrusion method, and a more aqueous solution was used for inkjet printing. Using extrusion printing, the authors prepared embedded tracks, for potential application as electrodes, by inserting the extruding needle under the surface of the biopolymer solution. The authors reported a higher conductivity for the extruded patterns compared to the inkjet printed ones, due to the greater amount of deposited conducting inks. Another study found that gellan gum is a good rheological modifier for MWNT inks [4]. It was found that a single layer of ink printed onto a flexible substrate exhibited resistance values of 7–8 kΩ/cm, which was reduced by increasing the number of printed layers. In recent years, organic dyes such as bile acid, cationic malachite green, and safranin are used as new dispersants for MWNTs [9,10,11].

Carrageenan is a large anionic biopolymer polysaccharide, which is highly flexible and form curly helical structure. It is extracted from red seaweeds and is known for its different gel forming properties at ambient temperature and used as good dispersants for CNTs, thickening and stabilizing agent [12,13]. There are three types of carrageenan based on number of sulphated group per biopolymer unit i.e., k-carrageenan (one sulphated group, KC), i-carrageenan (two sulphated groups, IC) and π-carrageenan (three sulphated groups, πC) [14,15]. Carrageenans are mainly employed in the food processing industry and for medical applications [16,17,18,19,20,21]. In food processing, carrageenans are used as water-based gels (such as cakes, desserts, and jellies), in dairy products (such as puddings and ice-cream), and as additives to improve the texture of cooked food. A research study added a mixture of KC and xanthan gum to mashed potatoes, and performed a variety of tests to study possible effects in food quality [17]. They found that, while the gum essentially acts as filler, carrageenan markedly improves parameters such as gel strength, viscosity, elasticity, and overall acceptability. As for their medical usage, carrageenans have been reported for their application in probiotic encapsulation, both as microcapsules and microspheres [18].

The present work investigates the influence of these two additives, that is i-carrageenan (IC) and glycerine (G) on the performance and properties of extrusion ink made of bucky paper CNT, particularly MWNT (multiwall carbon nanotube). Glycerine is a polyol and is used to increase the flexibility of polymer films and i-carrageenan is used to aid dispersion of CNT in aqueous medium. The composites inks containing CNTs (MWNT) and dispersants (IC and glycerine) were prepared through sonication, which were then characterized by rheology. The printed tracks were characterized using spectroscopy, surface morphology and electrical conductivity.

## 2. Materials and Methods

### 2.1. Materials

IC, mol. wt. from 350,000 to 800,000 g/mol, Genuvisco type C1-123, lot#SK93842) was obtained from CP Kelco, Atlanta, GA, USA. Glycerine (G, lot#033K0097) was procured from Sigma Aldrich, St. Louis, MI, USA. The structure of both carrageenan and glycerine are shown in supplementary section as Figures S1 and S2, respectively. Transparent polyethylene tetra-phthalate (PET) sheets were obtained from Corporate Express Sydney, Australia. Multiwall carbon nanotubes (MWNT, diameter = 9.5 ± 0.3 nm, length = 0.64 ± 0.17 μm), produced by chemical vapor deposition, were obtained from Nanocyl Incorporation, New York, NY, USA (lot#090901, P0348). Milli-Q water (resistivity, 18.2 MΩ cm) from Millipore Q water purification system, Merck Millipore, Ontario, Canada.

### 2.2. Methods

#### 2.2.1. IC and MWNT Dispersion

Initially, IC solution (1.5% *w*/*v*) was prepared by adding 1.5 mg of IC to 100 mL of Milli-Q water with stirring for 3 h at 70 °C on a hot plate using magnetic stir bar. Then homogeneous IC-MWNT dispersion solution (1 mg/mL) was prepared by sonication for 20 min using a digital sonicator (Branson 450, 400 W) with a probe of 10 mm diameter and power output of 12 W in pulse mode (0.5 s on-off). 10 mL unsonicated IC solution was added to sonicated dispersion solution to increase its viscosity and then mixed with Homozenizer (Wise Mix HG-15D, Witeg Labortechnik GmbH, Wertheim, Germany) at a speed of 3000 mp for 10 min. Thus, the non-Newtonian IC-MWNT ink was prepared for extrusion printing.

#### 2.2.2. Glycerine and MWNT Dispersion

Glycerine (G) solution 30% *v*/*v* was prepared by adding 4.5 mL of glycerine to 15 mL of Milli-Q water with stirring for 1 h at 50 °C. Glycerine-MWNT dispersion solution was made by adding 15 mg MWNT to the above solution, followed by sonication for 20 min using the above sonicator. The solution was heated in an oven to evaporate extra water to increase viscosity of the ink. The Newtonian G-MWNT ink was made ready for extrusion printing.

#### 2.2.3. Extrusion Printing

Ink IC-MWNT or G-MWNT was extruded using a syringe printer, (custom-built at the University of Wollongong, Wollongong, Australia). The printer consists of a gas pressure controller (EFD Ultimus I, under Nitrogen), connected to a syringe assembly (3 cc syringe with luer lock 100 µm stainless steel tips), strapped to the vertical axis of a CNC (computer numerical control) three-dimensional stage (Sherline 8020 CNC 8-Direction Vertical Mill, Sherline, Vista, CA, USA). Conductive patterns were printed onto glass (microscope slide), IC gel films (thickness = 45 µm) and transparent PET sheets.

## 3. Characterization

Rheological testing was conducted using a rheometer (Anton Paar–Physica, MCR 301) working with a probe head (diameter = 50 mm, cone 1° angle) at 21 °C and using Rheoplus (version 3.0X, Anton Paar GmbH, Graz, Austria) software. The temperature is controlled using a Peltier heating system and the motion of the instrument is controlled using compressed air. Carrageenan solutions and CNT dispersions were analyzed using flow curves (viscosity and shear stress vs. shear rate) and oscillatory strain sweeps to measure the dynamic modulus.

Surface morphology of the printed tracks (materials) was examined by using optical microscopy, scanning electron microscopy (SEM) and profilometry. The dispersions, and composite printed tracks, were imaged using an optical microscope (LEICA Z16 APO, Leica Microsystems, Mumbai, India) fitted with a digital camera (LEICA DFC280. Leica Microsystems, Mumbai, India). The images were acquired using the Leica Application Suite (version 3.1.0 R1, Leica Microsystems, Mumbai, India) software. Dispersions were dropped onto glass slides and printed tracks were images on their substrates. Images were typically acquired using 20× and 50× magnification.

SEM images were acquired using a JEOL JSM-7500FA, JEOL, Peabody, MA, USA. Samples were prepared by mounting small pieces of films onto a brass stub (11 × 5 mm^2^) using double–sided conductive carbon tape. When required, samples were coated with a thin platinum layer using an Edwards AUTO 306 Sputter system JEOL Peabody, MA, USA. For the printed tracks on various substrates, the thickness was determined using a contact profilometer (Veeco Dektak, Veeco Instruments Inc., Plainview, NY, USA) 150 with a 12.5 μm tip and 5.00 mg force.

The contact angles of all samples were measured using the sessile drop method and a goniometer (Data Physics SCA20, Carl Stuart Limited, Dublin, Ireland) fitted with a digital camera. The contact angles of 1 μL Milli-Q water droplets on the surface of the samples were calculated after 30 s using the accompanying Data Physics software (SCA20.1, Carl Stuart Limited, Dublin, Ireland). The mean contact angle was calculated based on the measurements of at least five water droplets.

The current (*I*)-voltage (*V*) characteristics and electrical resistance of the samples were evaluated using two-point and four-point probe techniques, respectively, under controlled conditions in air (21 °C, 45% relative humidity, RH) using a waveform generator (Agilent 33220A, Keysight Technologies, Santa Rosa, CA, USA) and a digital multimeter (Agilent 34410A, Keysight Technologies, Santa Rosa, CA, USA).

## 4. Results and Discussion

### 4.1. Rheology of Composite Inks

The flow characteristics of the composite inks were studied prior to the printing process. As with other printing apparatus, there are certain requirements for the viscosity of the ink; these requirements are set by the printing apparatus. For extrusion printing apparatus a much higher viscosity was required. For this reason, unsonicated IC was added to composite dispersions to increase the viscosity after sonication was completed. Further thickening of the dispersion was performed by evaporation of the water using an oven.

**Figure 1:**
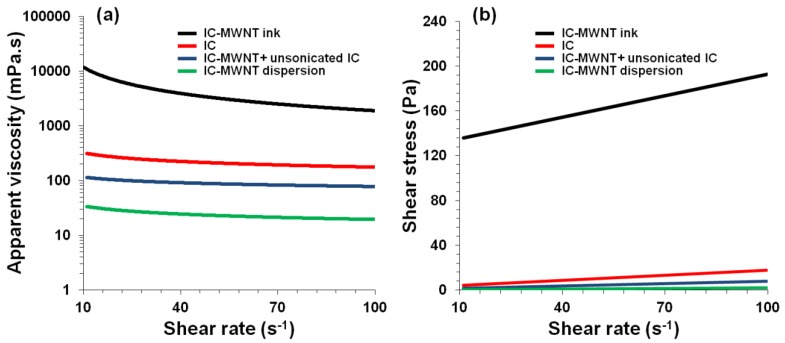
Flow curves for IC solution and IC-MWNT dispersions. (**a**) Apparent viscosity and (**b**) Shear stress as a function of shear rate for IC-MWNT ink after thickening process, IC solution (1.5% *w*/*v*), IC-MWNT after addition of unsonicated biopolymer and IC-MWNT composite dispersion. Lines in (**a**,**b**) are fits to Equations (1) and (2), respectively. All samples were measured at 21 °C.

**Figure 2:**
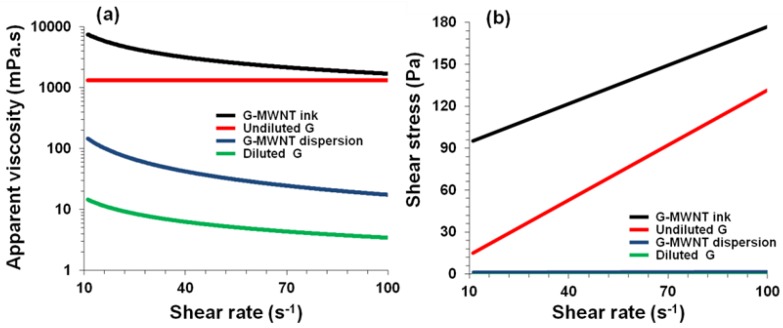
Flow curves for glycerin and G-MWNT dispersions. (**a**) Apparent viscosity and (**b**) Shear stress as a function of shear rate for G-MWNT ink after thickening process, undiluted glycerin, G-MWNT dispersion and diluted glycerin. Lines in (**a**,**b**) are fits to Equations (1) and (2), respectively. All samples were measured at 21 °C.

Flow curves were measured for each ink to gauge the viscosity and the effect of sonication and addition of (unsonicated) dispersant (Figure 1 and Figure 2 and Table 1). Both inks displayed shear-thinning behavior (Figure 1a and Figure 2a), i.e., the viscosity (η) decreases with increasing shear rate ($\dot{\gamma }$) which could be fitted to the well-known power-law model [3,22,23,24],

(1) $$\eta =K{\dot{\gamma }}^{n-1}$$

where *K* and *n* indicate the ‘consistency’ and power-law index, respectively. As expected, the viscosity decreased due to sonication and increased 4-fold upon addition of (unsonicated) dispersant. Furthermore, the decrease in apparent viscosity and consistency and the increase in the power-law index values due to sonication were reversed through addition of (unsonicated) dispersant.

The thickening process resulted in a 230-fold increase in the apparent viscosity (at shear rate 100 S^−1^) of the IC-MWNT ink (Figure 1a) and a 90-fold increase for G-MWNT ink (Figure 2a). Furthermore, the thickening process increased the ‘consistency’ of the dispersion from 172 ± 1 to 70,387 ± 49 mPa·s^n^ and reduced the power index value from 0.83 to 0.21. This suggests that the ink is becoming thicker (*K* increases) and more shear-thinning (*n* decreases).

The relation between shear stress (*τ*) and shear rate for IC-MWNT and G-MWNT inks is shown in Figure 1b and Figure 2b. It can be seen that the inks of varying concentration exhibit a yield point, i.e., these solutions only flow when a certain amount of force is applied. This point can be determined using modulus such as the Bingham model [3,25]:

(2) $$\tau =\tau_{B}+\eta_{B}\;\dot{\gamma }$$

where *τ_B_* and *η_B_* indicate the Bingham yield point and Bingham flow coefficient, respectively. Although the values obtained using the Bingham model are dependent on the shear rate range, it provides a good approximation for the determination of yield points [3].

The Bingham parameters were also increased due to this thickening process (Table 1). As with IC-MWNT ink, the G-MWNT ink had a dramatic increase in the consistency and power-law index, i.e., a 26-fold and 8-fold increase, respectively, which resulted in a thicker and more shear-thinning ink. It was shown that glycerine is a Newtonian fluid (n~1) indicating that its viscosity is independent of shear rate (Table 1). The addition of MWNTs imparts shear-thinning behavior, the ink becomes non-Newtonian.

### 4.2. Morphology of Extrusion Printed Tracks

#### 4.2.1. IC-MWNT Extrusion Inks

**Figure 3:**
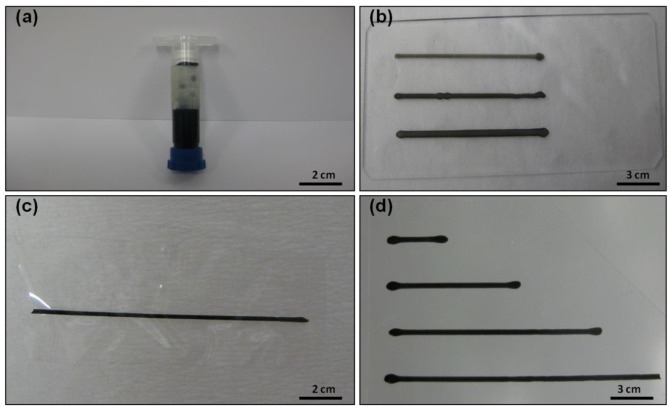
Photographs of IC-MWNT inks: (**a**) In syringe prior to printing; (**b**–**d**) printed tracks on glass (1, 2 and 3 layers thick), IC (2 layers) and PET (2 layers) substrates, respectively.

The composite inks were processed as solid conducting patterns by extrusion printing using a syringe (see Figure 3a) onto various substrates including microscope glass slides, PET sheets and IC films. An example of IC-MWNT inks printed onto each substrate is shown in Figure 3b–d.

**Figure 4:**
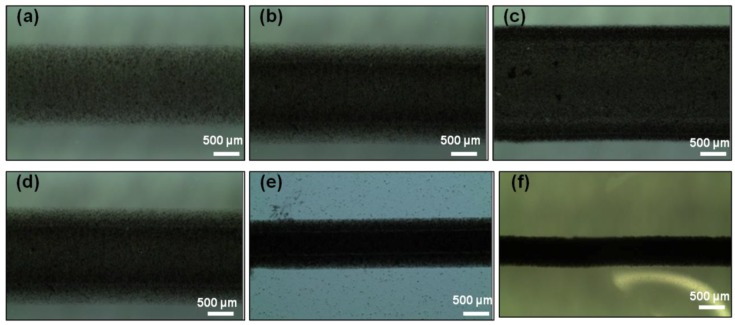
Microscopy images of IC-MWNT printed tracks: (**a**–**c**) 1–3 printed layers on microscope glass substrates and (**d**–**f**) 2 printed layers on glass, PET, and IC films substrates, respectively.

The surface morphology of the printed tracks as examined by using microscopy and profilometry are shown in Figure 4. Figure 4a–c, show that width of printed tracks increases with the number of layers. For example, the width of the printed patterns increased from 866 ± 11 µm for one layer to 974 ± 15 µm for two layers of IC-MWNT composite ink. However, upon printing two layers of the composite inks on the three different substrates (see Figure 4d–f); the width is visibly decreased from glass (974 ± 18 µm) to PET (567 ± 12 µm) to IC film (298 ± 8 µm) substrates. This corresponds with the increasing contact angles along with the absorbing nature of the IC film substrate.

**Figure 5:**
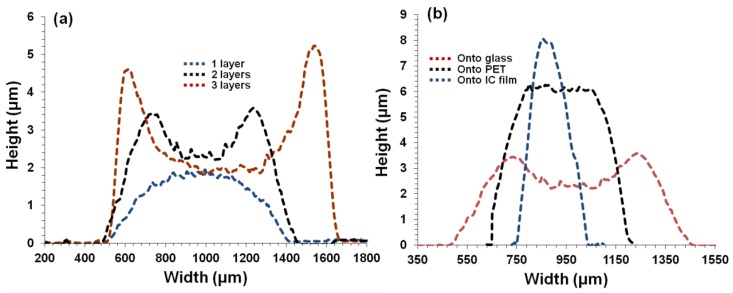
Profilometry curves of printed patterns IC-MWNT (**a**) 1–3 printed layers onto microscope glass substrates and (**b**) 2 printed layers onto glass, PET, and IC films substrates, respectively. Area under the curve represents the cross-sectional area.

**Figure 6:**
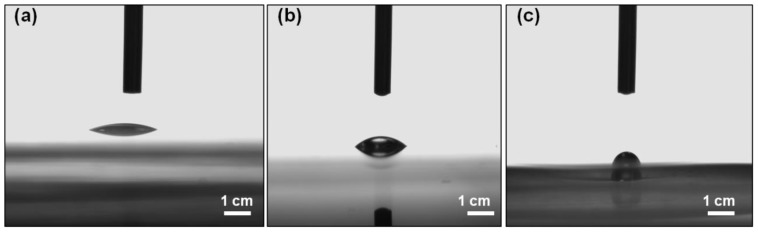
Contact angle measurement for water droplets on (**a**) glass slides; (**b**) PET and (**c**) IC film substrates.

Profilometry curves of printed patterns of IC-MWNT ink (see Figure 5a) show that increasing the number of extrusion printing layers causes an increase in the cross-sectional area, i.e., 1416 ± 80 µm^2^, 2541 ± 160 µm^2^ and 3200 ± 200 µm^2^ for 1, 2 and 3 layers, respectively. Furthermore, Figure 5b shows the profilometry curves of two layers of IC-MWNT composite inks printed on glass, PET, and IC film substrates. It is observed that the printed patterns on IC film substrates exhibited lower cross-sectional area, compared to that of the solid substrates i.e., 2108 ± 98 µm^2^ (IC film), compared to 2541 ± 154 µm^2^ (glass). This is attributed to part of the printed track staying below the IC film surface. It is also known that there is a difference in hydrophilicity for glass, PET, and IC film substrates. Thus, contact angles also vary for these substrates e.g., contact angles of water droplets for glass, PET and IC films are shown in Figure 6 and the values are 22° ± 1.8°, 34° ± 1.0° and 55° ± 2.1°, respectively. It is observed that the extruded tracks are becoming higher but narrower.

#### 4.2.2. G-MWNT Extrusion Inks

**Figure 7:**
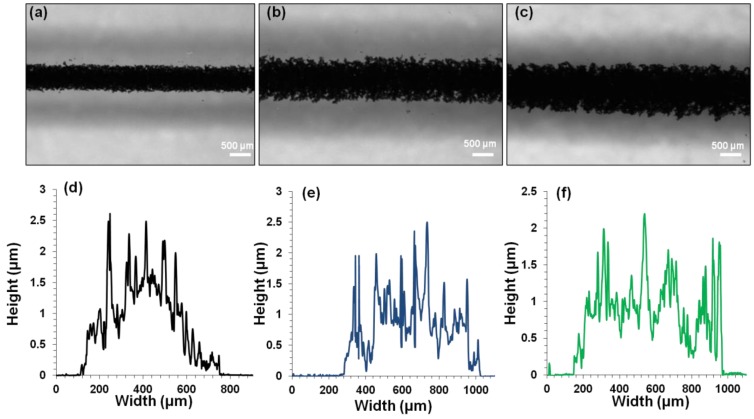
(**a**–**c**) Microscopy images of G-MWNT printed tracks 1, 2 and 3 printed layers on glass substrate. (**d**–**f**) Profilometry curves for 1, 2 and 3 layers of printed G-MWNT tracks on glass substrate. Area under the curve represents the cross-sectional area.

**Figure 8:**
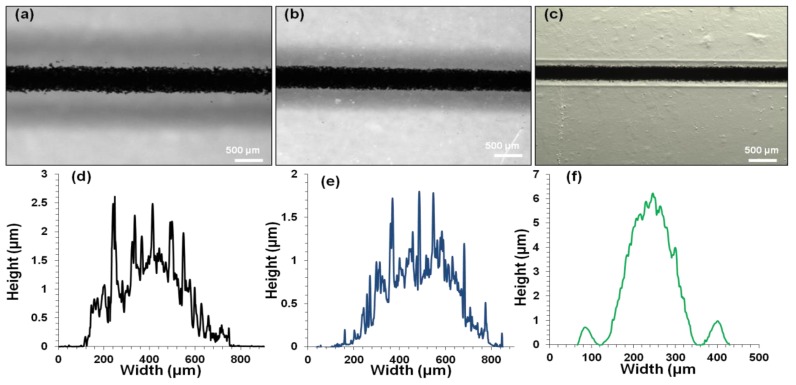
(**a**–**c**) Microscopy images and (**d**–**f**) profilometry curves of G-MWNT one printed layer on glass, PET, and IC film substrates, respectively. Area under the curve represents the cross-sectional area.

When the number of printed layers of G-MWNT inks onto glass substrate increased, the width of the tracks also increased, as seen in Figure 7a–c. For example, from 1 layer to 3 layers, the track width increased from 681 ± 9 µm to 825 ± 6 µm. In addition, the cross-sectional area also increased from 619 ± 12 µm^2^ to 752 ± 14 µm^2^ (Figure 7d–f). On the other hand, when G-MWNT inks were printed on different substrates such as glass, PET and IC film substrates, similar behavior to that of the IC-MWNT patterns was observed i.e., the width decreases from 681 ± 9 µm to 625 ± 12 µm to 229 ± 7 µm for glass, PET and IC film, respectively, as seen in Figure 8a–c. Cross-sectional profiles of G-MWNT extrusion ink printed onto glass, PET and IC film were measured from Figure 8d–f, the cross-sectional areas were found to be 619 ± 12 µm^2^, 438 ± 13 µm^2^ and 764 ± 19 µm^2^, respectively. Data show that the height of the track on the IC film substrate was four times greater than that observed for the glass substrate (6 and 1.5 µm, respectively). This discrepancy is related to the difference in contact angle between the two substrates which was seen in Figure 5 and the absorbing nature of the IC substrate, as mentioned previously. It seems that there appears to be a coating of water around the extrusion printing track of G-MWNT inks on IC film substrate (see Figure 8f). This could be because of water absorbance from the substrate to the ink. This may lead to the increase in the cross-sectional area to be 764 ± 19 µm^2^, as observed in Figure 8f.

### 4.3. SEM

**Figure 9:**
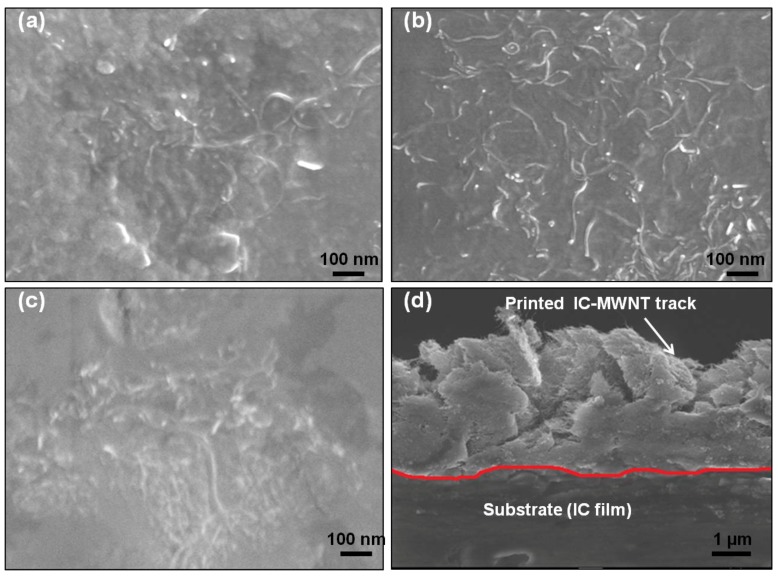
SEM images of IC-MWNT extruded patterns onto (**a**) glass; (**b**) PET sheet and (**c**) IC film substrates; (**d**) Cross-sectional view of IC-MWNT tracks printed onto IC film substrate. The red line indicates interface between track and the substrate.

**Figure 10:**
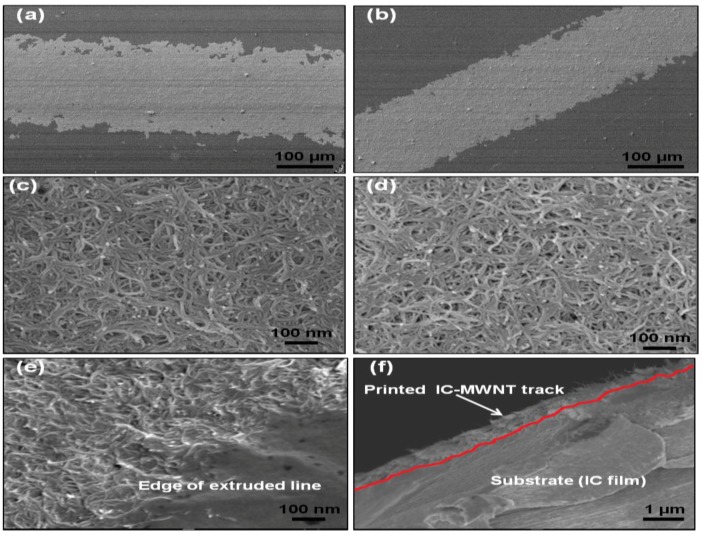
SEM images of G-MWNT extruded patterns onto (**a**,**c**) glass; (**b**,**d**) PET sheet and (**e**) IC film substrates; (**f**) Cross-sectional view of G-MWNT tracks printed onto IC film substrate. The red line indicates interface between track and the substrate.

The SEM images of IC-MWNT and G-MWNT printed tracks onto various substrates are shown in Figure 9 and Figure 10, respectively. As expected, there is a significant difference in the surface morphology between these two types of printed inks. MWNTs are clearly visible in the tracks printed with glycerine inks. Figure 9d and Figure 10f show cross sections of the IC-MWNT and G-MWNT printed tracks onto an IC film substrate, respectively. Part of the ink is visibly embedded into the surface of the IC film substrate. This phenomenon has been previously observed for composite inks printed onto a similar biopolymer’s substrate (chitosan and gellan gum) [3,4].

Edge of the G-MWNT extrusion ink printing tracks on glass appear to be fractured or discontinuous, whereas the G-MWNT inks printed tracks on PET appear to be smoother. This may be attributed to the difference in hydrophobicity of the two substrates (Figure 6), which leads to a less wetting on PET compared to glass.

### 4.4. Electrical Conductivity of Printed Tracks

**Figure 11:**
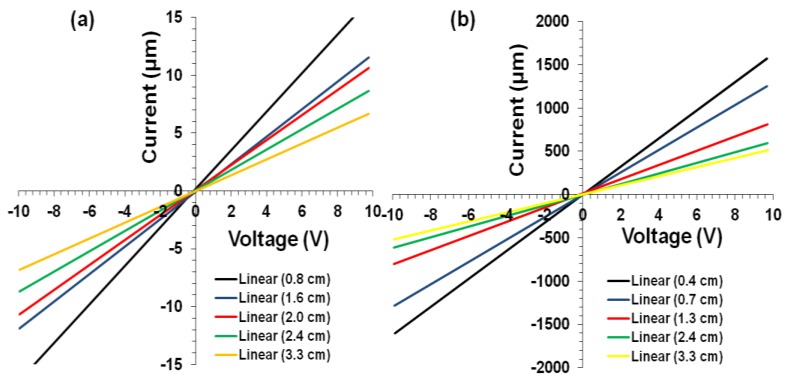
Current-voltage (I-V) plots obtained using five different lengths for (**a**) IC-MWNT and (**b**) G-MWNT inks printed on glass substrate.

Electrical resistance measurements were obtained for IC-MWNT and G-MWNT tracks on various substrates (glass, PET, and IC film). I-V characteristics were investigated under controlled conditions (21 °C, RH = 45%). All extruded tracks exhibited linear I-V characteristics indicating Ohms behavior (Figure 11).

**Figure 12:**
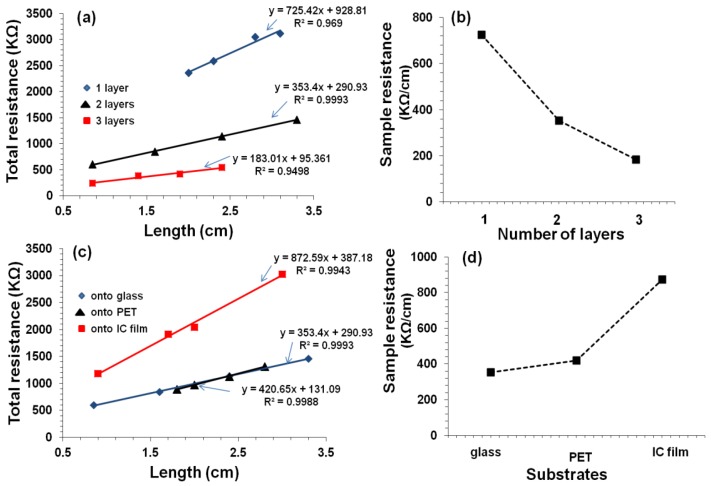
Resistance vs. length for extruded patterns of IC-MWNT ink. (**a**) 1–3 printed layers onto microscope glass substrates; (**b**) sample resistance of 1–3 printed layers on microscope glass substrates, derived from the slope of the lines in (**a**); (**c**) Resistance vs. length for extruded two layers patterns on glass, PET and IC films substrates; (**d**) Sample resistance of the tracks printed onto different substrates, derived from the slope of the lines in (**c**).

**Figure 13:**
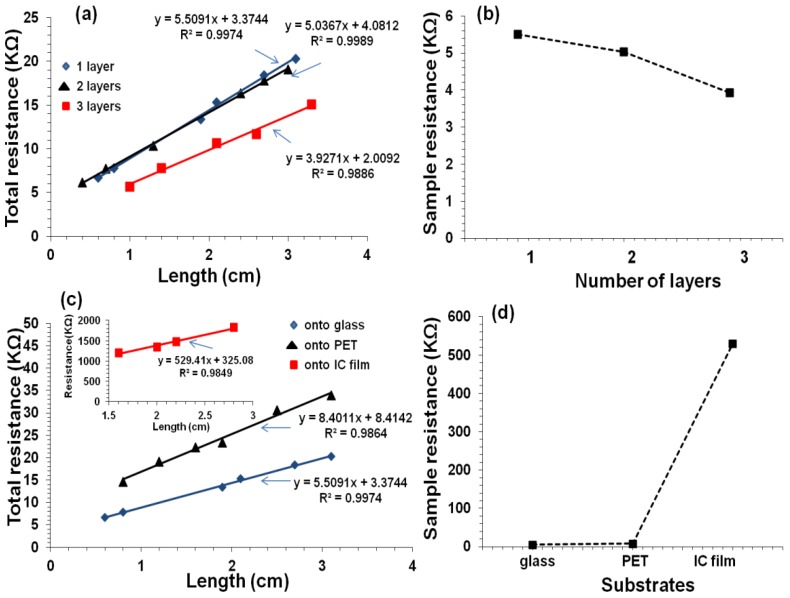
Resistance vs. length for extruded patterns of G-MWNT ink. (**a**) 1–3 printed layers on microscope glass substrates and (**b**) sample resistance of 1–3 printed layers on microscope glass substrates, derived from the slope of the lines in (**a**); (**c**) Resistance vs. length for two printed layers on glass, PET, and IC films (inset) substrates. The inset in (**b**) is present due to the vast different in y-axis values; (**d**) Sample resistance of the tracks printed onto different substrates, derived from the slope of the lines in (**c**).

When number of printed layers is increased, electrical resistance decreased i.e., the track resistance decreased from 725 kΩ/cm for one layer to 183 kΩ/cm for three layers (Figure 12). It is well known that when CNT concentration is increased by overprinting, the electric pathways also increase, which decreases the resistance [25,26]. It is also noted that the contact resistance decreased by an order of magnitude when the number of printed layers increased from 1 to 3. This could be attributed to an increase in the electrode-film contact area. It was observed with two layers of IC-MWNT printing on glass and IC film, the resistance increased from 353 to 872 kΩ/cm for glass and IC film, respectively. This increase can be explained using the SEM images (Figure 10f), which revealed that part of the printed track lay beneath the surface of the IC film substrate. Similar observations were obtained for G-MWNT extruded tracks after increasing the number of layers and changing the type of substrate (see Figure 13a–d).

Conductivity of the extruded tracks was evaluated, and results are shown in Table 2. As expected, the conductivity was found to increase from 9 ± 1 S/m to 17 ± 3 S/m when the number of extruded layers of IC-MWNT composite ink on glass substrate increased from 1 to 3. This trend was also observed in previous studies with printing CNT composite inks [1,25]. It was also found that the substrate played an important role in electrical conductivity of extruded tracks (see Table 2). For example, changing the substrate from glass to an IC free-standing film, conductivity decreased by 55% for two extruded layers. This effect of increasing the conductivity by increasing the number of layers agrees with previous results [3,4]. The conductivity of G-MWNT extrusion ink printing tracks was found to be three orders of magnitude higher than that of IC-MWNT, i.e., 3400 S/m. This could be attributed to the molecular weight difference between IC biopolymer (350,000–800,000 g/mol) and glycerine molecules (92.09 g/mol). The IC biopolymer results in an almost entirely coated MWNT network; hence the conductivity was reduced. This can be attributed to the fact that the conductivity within the carrageenan/CNT composite system is due to the formation of conductive continuous network of conductive additive CNTs within the carrageenan matrix [27,28,29]. The charge carriers present within the CNTs flow through this continuous conductive networks and account for its conductivity. As the biopolymer carrageenan is insulating in nature, hence the increase in its amount within the composite make hindrance in the flow of charge carriers present in CNTs and thereby drop in conductivity is observed. The difference in CNTs coating between IC and glycerine is clearly visible in the SEM images (Figure 9 and Figure 10).

## 5. Conclusions

Extrusion printing methods were used to fabricate conducting patterns of MWNT composite dispersions. Extrusion printing gives tracks of higher electrical conductivity due to higher deposition rate. It was also found that the substrate materials played an important role in the width and cross-sectional area of the printed tracks. Solid substrates (glass) resulted in a larger width and cross-sectional area, compared to the narrower but higher tracks printed onto IC films. This is due to the absorbing nature of the IC film and the difference in hydrophobicity between the substrates. Extrusion printing inks require that IC-MWNT and G-MWNT composite inks should have higher viscosities and therefore a thickening process was used to increase the viscosities to 1880 and 1720 mPa·s, respectively.

The electrically conducting tracks produced by extrusion printing, using IC and glycerine as dispersants, were compared. The conductivity of IC-MWNT tracks was 9 ± 1 S/m, compared to 2942 ± 84 S/m for G-MWNT tracks printed onto a glass substrate of one layer thick. This increased conductivity is attributed to the lesser degree of CNT coating in the G-MWNT matrix, as observed in SEM images. The nature of the substrate was found to affect the electrical conductivity of printed patterns. A two orders of magnitude increase was observed for the conductivity of G-MWNT printed onto glass substrate compared to IC film substrates. This method of extrusion printing with Newtonian fluid to produce CNT conducting tracks has not been reported previously. The obtained conductivity (3400 S/m) is approaching the upper limit of MWNT films (~5500 S/m), which is an impressive result. The results show that the developed conducting materials may function as flexible, biocompatible conductors for implanted sensors. Moreover, the printing of composite materials into conducting tracks could be adapted for the fabrication of intricate circuits for flexible electronic devices.

## Figures and Tables

**Table 1:** Summary of rheological analysis at a shear rate range of 10–100 s^−1^ and 21 °C for printed IC-MWNT and G-MWNT inks. Concentrations of IC, CNT and G are 1.5% *w*/*v*, 0.10% *w*/*v* and 0.38% *w*/*v*, respectively Consistency (*K*) and power-law index (*n*) values were obtained through curve fitting with the power-law model (Equation (1)). Bingham yield point (*τ_B_*) and Bingham flow coefficient (*η_B_*) values were obtained using the Bingham model (Equation (2)).

Sample	*K* (mPa·s^n^)	*n*	*τ_B_* (Pa)	*η_B_* (Pa.s)
IC	585 ± 2	0.74 ± 0.01	2.60 ± 0.13	0.152 ± 0.002
IC-MWNT	60.90 ± 1.93	0.75 ± 0.01	0.22 ± 0.01	0.018 ± 0.001
IC-MWNT + IC	172.19 ± 0.36	0.83 ± 0.01	0.73 ± 0.04	0.071 ± 0.001
IC-MWNT ink	70,387 ± 49	0.21 ± 0.02	128.79 ± 0.83	0.640 ± 0.013
G	1320 ± 0.1	0.99 ± 0.01	0.19 ± 0.02	1.314 ± 0.002
Water-G	70.15 ± 0.84	0.34 ± 0.02	0.15 ± 0.01	0.002 ± 0.001
G-MWNT	1474 ± 17	0.04 ± 0.02	1.38 ± 0.01	0.005 ± 0.001
G-MWNT ink	37,965 ± 45	0.32 ± 0.01	85.05 ± 0.98	0.918 ± 0.016

**Table 2:** Summary of electrical conductivity (σ) values of IC-MWNT and G-MWNT composite inks extruded on various substrates (glass, PET, and IC film).

Ink	Number of Layer	Substrate	σ (S/m)
IC-MWNT	1	Glass	9 ± 1
IC-MWNT	2	Glass	11 ± 2
IC-MWNT	3	Glass	17 ± 3
IC-MWNT	2	PET	8 ± 1
IC-MWNT	2	IC film	5 ± 1
G-MWNT	1	Glass	2942 ± 84
G-MWNT	2	Glass	3095 ± 71
G-MWNT	3	Glass	3384 ± 85
G-MWNT	1	PET	2679 ± 90
G-MWNT	1	IC film	24 ± 6

## Supplementary Materials

The following are available online at http://www.mdpi.com/2073-4360/10/10/1148/s1, Figure S1: Chemical structure of the three main types of carrageenan (**a**) iota, (**b**) kappa and (**c**) lambda., Figure S2: Chemical structure of glycerine.
