# Supplementary material for: Influence of Biopolymer Carrageenan and Glycerine on the Properties of Extrusion Printed Inks of Carbon Nanotubes
Source: Polymers (Basel). 2018 Oct 15;10(10):1148. doi: 10.3390/polym10101148 (PMC6404002; doi:10.3390/polym10101148)
Supplement: Supplementary file 1 [file polymers-10-01148-s001.pdf]

Polymers 2018, 10, 1148; doi:10.3390/polym10101148 www.mdpi.com/journal/polymers

polymers MDPI

Supplementary Information: Influence of Biopolymer Carrageenan and Glycerine on the Properties of Extrusion Printed Inks of Carbon Nanotubes

Mohammed Almoiqli[1], Ali Aldalbahi[2*], Mostafizur Rahaman[2*], Periyasami Govindasami[2], Shaykha Alzahly[3]

[1] Nuclear Sciences Research Institute, King Abdulaziz City for Science and Technology, Riyadh 11442, Saudi Arabia; almoiqli@kacst.edu.sa (M.A.)
[2] Department of Chemistry, College of Science, King Saud University, Riyadh 11451, Saudi Arabia; pkandhan@ksu.edu.sa (P.G.)
[3] King Abdullah Institute for Nanotechnology, King Saud University, Riyadh 11451, Saudi Arabia; shaykha.alzahly@hotmail.com (S.A.)

* **Correspondence:** aaldalbahi@ksu.edu.sa (A.A.); mrahaman@ksu.edu.sa (M.R.); Tel.: +966-114-675-883

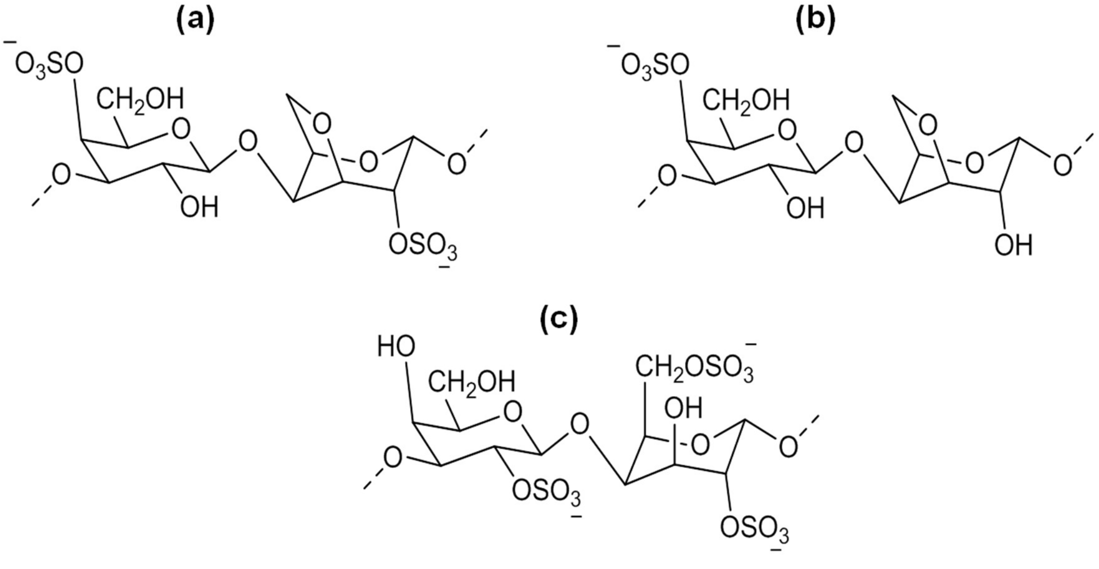

Figure S1. Chemical structure of the three main types of carrageenan (a) iota, (b) kappa and (c) lambda.

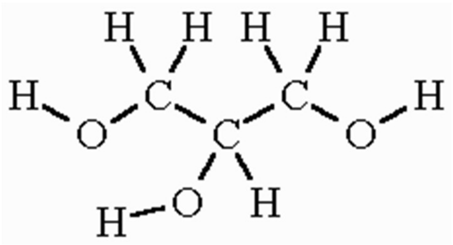

Figure S2. Chemical structure of glycerine.
